# Two- and three-dimensional sonographic findings of harlequin ichthyosis: case report and literature review^☆^

**DOI:** 10.1016/j.abd.2022.09.013

**Published:** 2023-06-22

**Authors:** Zesi Liu, Chunli Jing

**Affiliations:** a Department of Gynecology and Obstetrics, The First Affiliated Hospital Dalian Medical University, Dalian, China; b Department of Ultrasound of Gynecology and Obstetrics, The Second Affiliated Hospital Dalian Medical University, Dalian, China

**Keywords:** Ichthyosis, lamellar, Ultrasonography, Ultrasonography, prenatal

## Abstract

**Background:**

Harlequin ichthyosis (HI) is a rare skin disorder with extremely high lethality due to a mutation of the ABCA12 gene. Because of its rarity and the often-late onset, prenatal screening for HI is extremely difficult, and most pregnant women might easily miss the period for optimal examinations.

**Objective:**

To summarize the sonographic features of HI for prenatal diagnostic purposes.

**Methods:**

The authors describe a case of HI with no family history who was diagnosed by using prenatal ultrasound scanning. The sonographic features of HI and the clinical characteristics of pregnant women were summarized by searching relevant literature over nearly two decades.

**Results:**

The unique sonographic presentations including peeling skin, clenched hands and clubfeet, ectropion, flat nose, fetal growth impairment, polyhydramnios and echogenic amniotic fluid may be primarily related to skin disorders in HI fetuses. The authors also identified a novel pathogenic ABCA12 gene mutation and explained the possible pathogenic mechanisms.

**Study limitations:**

Caution should be exercised in summarizing disease characteristics because of the small number of cases, and the authors are faced with the possibility of incomplete case searching.

**Conclusions:**

HI has relatively unique sonographic features. Therefore, 2D-ultrasound combined with 3D-ultrasound may be an effective method for the prenatal diagnosis of HI. Moreover, a novel pathogenic ABCA12 gene mutation may provide important clues for future research on the etiology of HI. However, the authors consider that additional studies are needed to provide more evidence for prenatal diagnosis.

## Introduction

Harlequin ichthyosis (HI) is a rare autosomal recessive genetic disease (prevalence 1/300,000 births) with a high mortality rate.^1^, ^2^ Fetuses with HI can develop severe skin disorders during development, including large-thick, plate-like scales covering the whole body, eclabium, severe ectropion, and fattened ears, and late progression to severe scaling erythroderma. Respiratory failure, loss of fluid and heat, skin infections, and electrolyte metabolic disorders are the key reasons for the poor prognosis of HI. Unfortunately, there is no effective treatment for HI.^3^ Reliable methods for prenatal diagnosis are therefore essential for neonatal HI.

Prior to 2005, no candidate genes for HI have been identified and the diagnosis of HI relied on intrauterine fetal skin biopsies at 19‒23w.^4^, ^5^, ^6^ However, because this method is invasive and technically difficult, it increases the risk of multiple adverse pregnancy outcomes. Therefore, after the gene ABCA12 was identified as the causative gene for HI, ABCA12 mutational analysis by testing amniotic fluid and umbilical cord blood became an effective method for prenatal diagnosis of HI.^7^, ^8^ However, pregnant women with no family history or previous history of HI pregnancy, often do not choose to undergo HI-specific prenatal screening using the methods mentioned above. Ultrasound, however, as a non-invasive and inexpensive test, appears to be an effective method for prenatal diagnosis of HI.

In this study, the authors successfully performed prenatal diagnosis using 2D combined with 3D ultrasound in a fetus with HI with no family history. Chromosomal Microarray Analyses (CMAs) and Sanger sequence analysis were used to identify fetal chromosome abnormalities and obtain a pathogenic ABCA12 gene mutation.^9^, ^10^ In addition, by searching the published literature on HI, the authors summarized the sonographic features of HI.

## Methods

### Data sources

The ultrasonographical and clinical data of the patient were obtained based on the electronic medical database of the Second Affiliated Hospital of Dalian Medical University. The GE Voluson E8 color ultrasound diagnostic instrument with the probe frequency set from 2.5‒5.0 MHz was used in the present study. MicroRNA microarray analysis was processed with a commercial 750 K microarray chip and mutations were detected by Sanger sequence data analysis.

### Search strategy

The search strategy in MEDLINE/Pubmed used the following keywords: “Ichthyosis, Lamellar” and “Ultrasonography, Prenatal” [Mesh], adapted to the other databases when necessary.

## Results

### Case presentation

The present case is of a 40-year-old woman, gravida 1, para 0, conceived spontaneously with no family history. The mother denied medication history or infection and a history of adverse environmental exposure during pregnancy. Ultrasound findings at 13-week early pregnancy screening and ultrasound findings at 24^+3^-week middle pregnancy screening showed no significant abnormalities and Nuchal Translucency (NT) was 2.1 mm. The results of Noninvasive Prenatal Screening (NIPS) were negative. Until 30^+2^-week of pregnancy, other prenatal examination results were normal.

**Figure 1:**
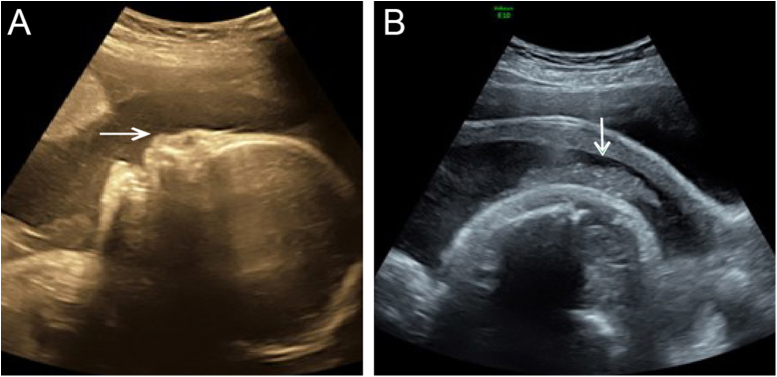
(A) 2-D ultrasound: The fetal face shows a flat curve and nasal hypoplasia (arrow) at the median sagittal section. (B) 2-D ultrasound: Abnormal skin flocculent echo (arrow)

**Figure 2:**
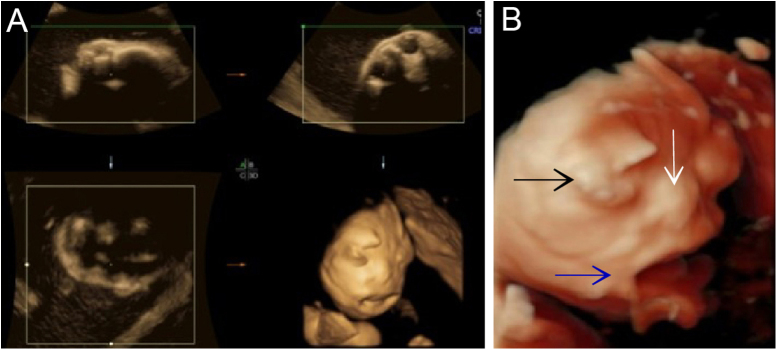
(A) 3-D ultrasound: the image of the face of the fetus. (B) 3-D ultrasound: eyelid edema and ectropion (black arrow); Hypoplastic nose (white arrow); Eclabium and anterior tongue protraction (blue arrow)

**Figure 3:**
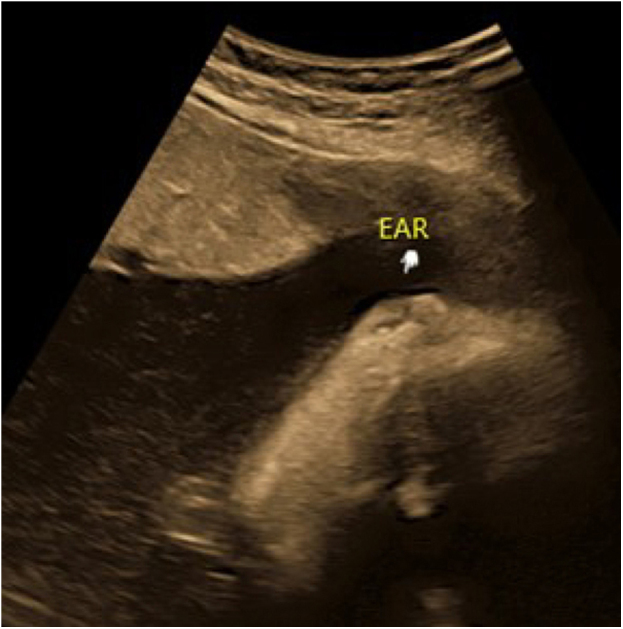
2-D ultrasound: microtia (arrow)

Although the ultrasound examination in the 30^+2^-week late pregnancy suggested that no significant development indicators of abnormality were noted (Table 1), the authors observed typical sonographic features, including a flat nose, peeling skin (Fig. 1A, B), ectropion, the tongue was upturned and abnormally large mouth cleft (similar to fish mouth) (Fig. 2A, B), microtia (Fig. 3) and Amniotic Fluid Index (AFI) was 340 mm. Then, US evaluation of other organs such as the brain, heart, and kidneys was normal and CDFI indicated that there is no obvious abnormality of the middle *cerebral artery* and umbilical artery blood flow signal.

**Table 1:** Ultrasound measurement of development parameters

Development indicators	BPD	HC	AC	FL	HL	EFW	EGW
This case	75 mm	277 mm	252 mm	54 mm	49 mm	1373 ± 200 g	29 + 5 w

BPD, Biparietal Diameter; HC, Head Circumference; AC, Abdominal Circumference; FL, Femur Length; HL, Humerus Length; EFW, Estimated Fetal Weight; EGW, Estimated Gestational Week.

**Figure 4:**
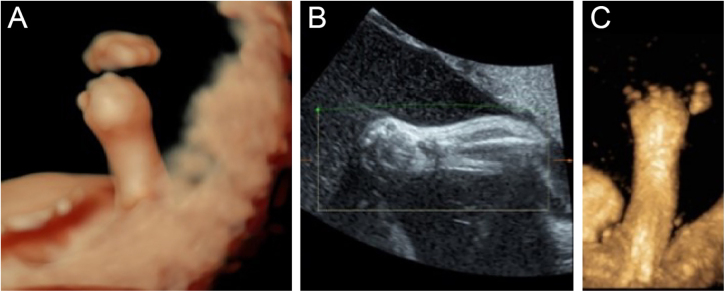
(A) 3-D ultrasound: clenched hands and skin oedema. (B) 2-D ultrasound: a shortening of phalanges and metacarpals. (C) 3-D ultrasound: the image of the upper limb of the fetus

**Figure 5:**
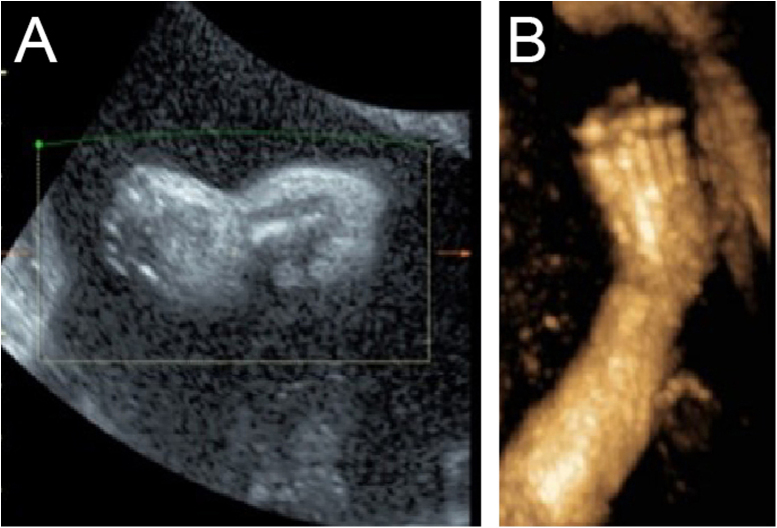
(A) 2-D ultrasound: the image of the right feet of the fetus. (B) 3-D ultrasound: pedal oedema and clubfeet

**Figure 6:**
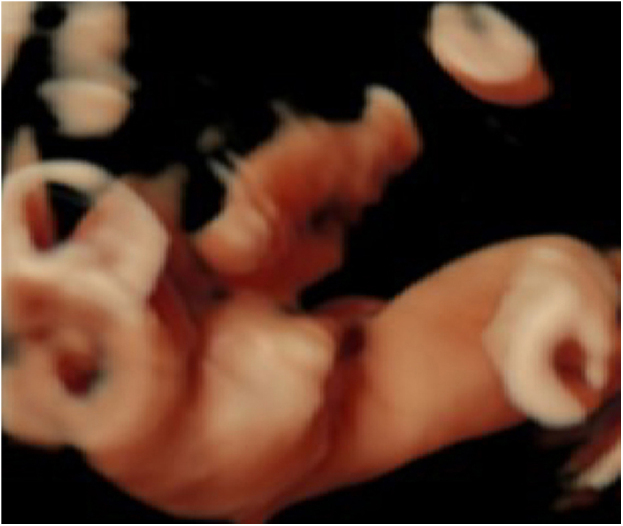
3-D ultrasound: no testicles seen in scrotum

**Figure 7:**
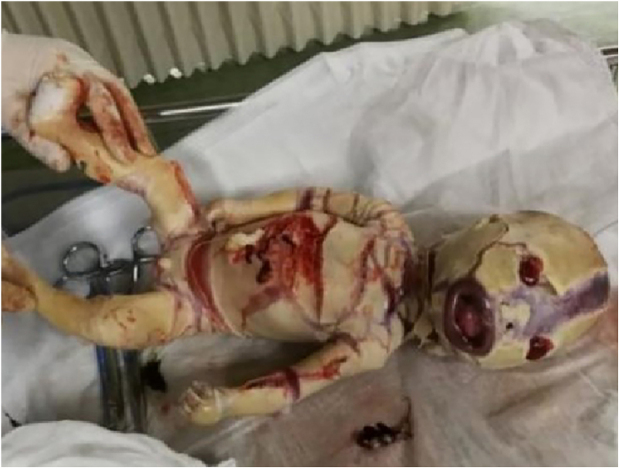
The appearance of abortus with Harlequin Ichthyosis was consistent with our prenatal ultrasound diagnosis

For better observation, Three-Dimensional (3D) image reconstruction was carried out and the findings are as follows: palms edema, clenched hands, a shortening of phalanges and metacarpals (Fig. 4A‒C), clubfeet (Fig. 5A, B), undescended testis (Fig. 6) and echogenic amniotic fluid. Subsequent to counseling, the pregnant couple decided to *terminate* the pregnancy and genetic testing was ordered. The skin of the fetus was thickened, with deep fissures between the skin and erythema skin spread throughout the *body,* resembling an “armor”. There was ectropion, a flat and small nose. The mouth opening and tongue protrusion were observed. Moreover, limbs were fixed and edematous, the hands were clenched, the feet were turned inward, and the little fingers and toes were small. The testes were also not descended. It was confirmed that the diagnosis based on prenatal ultrasound was accurate (Fig. 7).

### Chromosomal microarray assays and sanger sequence analysis

**Figure 8:**
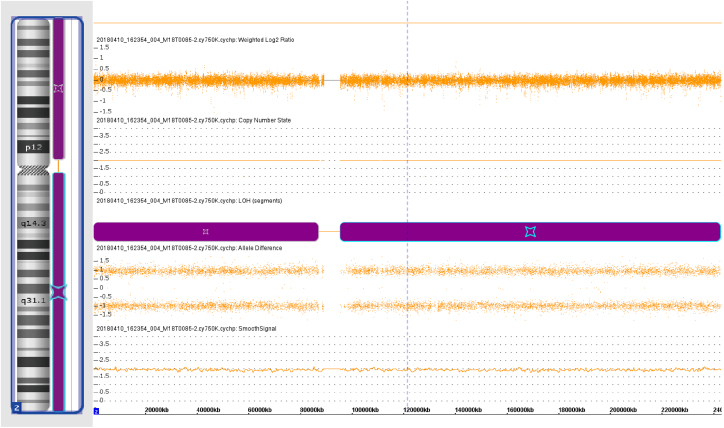
Microarray result displays for chromosome 2

**Figure 9:**
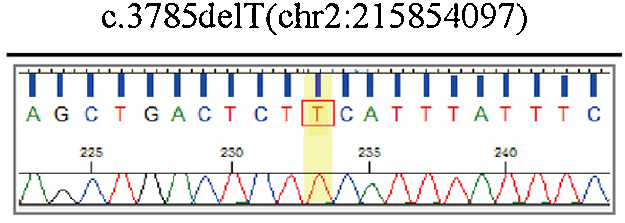
Sequence analysis performed in this case identified a novel pathogenic ABCA12 gene mutation ‒ ABCA12:c.3785delT and the mutations is a frameshift and deletion mutation, which lead to premature termination codons.

The preliminary results of chromosomal *microarray* assays suggested that genotype information revealed loss *of heterozygosity* (LOH) in chromosome 2 (Fig. 8). Furthermore, a pathogenic ABCA12 gene mutation - ABCA12:c.3785delT in homozygosity was identified based on the result of Sanger sequence analysis (Fig. 9).

### Literature search

Through searching the published cases, the authors identified 13 cases of HI diagnosed by prenatal ultrasound. By integrating clinical features and ultrasound sonographic characteristics of all cases, the authors found that the maternal age fluctuated in a wide range in 27‒40 years and that the proportion of patients with a family history was low (3/13). The onset of HI was concentrated in the third trimester of pregnancy and none of the HI fetuses survived (Table 2).^11^, ^12^, ^13^, ^14^, ^15^, ^16^, ^17^, ^18^, ^19^, ^20^ The authors have analyzed and summarized 13 typical ultrasonic characteristics of HI (Table 3).^11^, ^12^, ^13^, ^14^, ^15^, ^16^, ^17^, ^18^, ^19^, ^20^ Peeling skin, clenched hands and clubfeet, ectropion, flat nose, fetal growth restructuration, polyhydramnios, and echogenic amniotic fluid are more frequent (≥ 5). In addition, with this case, the authors found HI fetuses with combined microtia, a shortening of phalanges and metacarpals, and genital anomalies (undescended testis), which have rarely or never been mentioned in previous reports.

**Table 2:** Clinical characteristics and pregnancy outcomes of all cases

ID	Age	Precentage	Gestational week of ultrasonography	Family history or HI maternal outcomes	Pregnancy outcomes
Case 1^11^	31	G3P0	31w	No	Death
Case 2^11^	28	G1P0	30w	No	Death
Case 3^12^	–	G3P2	–	HI maternal outcomes	Death
Case 4^13^	35	G1P0	31w	No	Death
Case 5^14^	34	G1P0	31 + 5 w	No	Death
Case 6^15^	27	G2P1	27w	No	Death
Case 7^16^	–	–	–	–	Death
Case 8^17^	–	G2P1	–	–	Death
Case 9^18^	–	G1P0	34w	No	Death
Case 10^18^	30	G5P3	33w	No	Death
Case 11^18^	27	G3P2	27w	HI maternal outcomes	Death
Case 12^19^	34	G1P0	32w	No	Death
Case 13^20^	32	G2P0	30w (First time)/25 w (Second time)	HI maternal outcomes	Death
This case	40	G1P0	30 + 2 w	No	Death

The symbol “–” indicates the data previously not mentioned in the literature.

HI, Harlequin Ichthyosis.

**Table 3:** Thirteen typical sonographic characteristics of HI from published cases

Ultrasonographic Findings	Ectropion	Clenched hands and clubfeet	Peeling skin	Flat nose	Fetal growth restriction	Polyhydramnios
Case 1^11^	+	+	+	+	–	–
Case 2^11^	+	–	+	+	+	–
Case 3^12^	+	+	–	+	+	+
Case 4^13^	+	+	+	+	–	–
Case 5^14^	+	+	+	+	+	–
Case 6^15^	+	+	+	+	–	–
Case 7^16^	+	–	+	+	–	–
Case 8^17^	+	–	–	–	–	–
Case 9^18^	+	+	+	–	+	+
Case 10^18^	+	+	+	–	+	+
Case 11^18^	+	+	+	–	+	+
Case 12^19^	–	+	+	+	–	+
Case 13^20^	–	+	–	+	+	–
This case	+	+	+	+	–	+
Total	12	11	11	10	7	6

Ultrasonographic Findings	Echogenic amniotic fluid	Fish mouth	Macroglosssia	Subcutaneous edema	Micrognathia	Microcephaly	Short umbilical cord
Case 1^11^	+	+	–	–	–	–	–
Case 2^11^	+	–	–	+	–	–	–
Case 3^12^	–	–	–	–	+	–	+
Case 4^13^	–	+	–	–	–	–	–
Case 5^14^	+	+	+	+	+	+	–
Case 6^15^	–	–	–	–	–	–	–
Case 7^16^	–	–	+	–	–	–	–
Case 8^17^	–	–	–	–	–	–	–
Case 9^18^	–	–	–	–	–	–	–
Case 10^18^	–	–	–	–	–	–	–
Case 11^18^	–	–	–	–	–	–	–
Case 12^19^	+	–	+	–	–	–	–
Case 13^20^	–	–	–	–	–	+	–
This case	+	+	+	+	–	–	–
Total	5	4	4	3	2	2	1

The symbols “+” or “−” represent presence or absence sonographic features respectively.

## Discussion

HI is a rare genodermatosis with high lethality, and there is a lack of effective treatment for the condition. Based on the results of this study, the authors found a wide maternal age range of HI cases (ranging from 27‒40 years) and no significant abnormalities in early and mid-trimester examinations. Because of its rarity and the time of onset, prenatal screening for HI is extremely difficult and most pregnant women, especially those without a family history, might be easily missed the window for optimal examinations such as amniotic fluid and umbilical cord blood molecular detection.^11^ However, some unique sonographic features of HI could be detected in late pregnancy (ranging from 27w‒34w) ultrasound screening. Prenatal use of ultrasound imaging, because of the lack of ionizing radiation, convenience and clinically applicable, is expected to be an effective tool for prenatal screening for HI.

In this study, the authors summarized the major sonographic characteristics of HI by searching the relevant literature and the results were reported in Table 3.^11^, ^12^, ^13^, ^14^, ^15^, ^16^, ^17^, ^18^, ^19^, ^20^ The features with the occurrence of more than 5 were as follows: (1) Peeling skin; (2) Clenched hands and clubfeet; (3) Ectropion; (4) Flat nose; (5) Fetal growth restructuration; (6) Polyhydramnios; (7) Echogenic amniotic fluid. The authors believe that the unique sonographic presentations described above may be primarily related to skin disorders in HI fetuses. For example, the abnormal thickening of the skin may limit the movement of the fetal limbs and affect intrauterine growth and development. Since the amniotic fluid and fetal skin undergo free diffusion during pregnancy,^21^ this also seems to explain why HI can cause polyhydramnios. Peeling skin is also considered to be one of the causes of echogenic amniotic fluid.

Three-dimensional observation enabled us to get more visual information from stereo images and to describe some sonographic characteristics of HI. In addition, since most HI may have polyhydramnios, which is more favorable for three-dimensional image acquisition.^22^ Therefore, the authors argue that a combination of 2D and 3D ultrasound may be more beneficial for the prenatal diagnosis of HI.

ABCA12, localized to the cell membrane, is an epidermal keratinocyte lipid transporter and a defect in ABCA12 leads to skin lipid barrier breakdown.^8^, ^23^ The results of the chromosome microarray of this case showed the Loss of Heterozygosity (LOH) in chromosome 2. Sequencing analysis of gene ABCA12 identified a novel mutation ‒ ABCA12:c.3785delT. The ABCA12:c.3785delT mutation type is a frameshift and deletion mutation that causes phenylalanine converts to serine in its coding peptide chain and generates a premature termination codon at position 42 of the new reading frame. Carrying premature stop codons can yield truncated proteins^24^ and induce an mRNA degradation process called nonsense-mediated mRNA decay.^25^ These causes led to the loss of function of the original protein (p.Phe1262Serfs*42). The findings of this case provide new clues for further investigation into the pathogenesis of HI.

## Conclusion

HI has relatively unique sonographic features, therefore, systematic examination of pregnant women using 2D-ultrasound combined with 3D-ultrasound in the third trimester of pregnancy can sharply reduce the rate of missing diagnosis or misdiagnosis of HI. Moreover, the authors identified a novel pathogenic ABCA12 gene mutation and explained possible pathogenic mechanisms.

## Financial support

None declared.

## Authors’ contributions

Zesi Liu: Approval of the final version of the manuscript; critical literature review; effective participation in research orientation; preparation and writing of the manuscript; study conception and planning.

Chunli Jing: Approval of the final version of the manuscript; effective participation in research orientation; data collection, analysis, and interpretation; critical review of the manuscript; study conception and planning.

## Conflicts of interest

None declared.
